# Cardiovascular Risk Factors Among Younger and Older C-AYA Cancer Survivors Treated with Anthracyclines: A Single-Center Analysis

**DOI:** 10.3390/cancers18010012

**Published:** 2025-12-19

**Authors:** Matthew Dean, Ben Bane, OreOluwa Aluko, Yiwei Hang, Ericka Miller, Sherin Menachery, David Chuquin, Adam Aston, Xiaoyan Deng, Dipankar Bandyopadhyay, Jennifer Jordan, Uyen Truong, Madhu Gowda, Wendy Bottinor

**Affiliations:** 1 Internal Medicine Residency, Virginia Commonwealth University, Richmond, VA 23298, USA; matthew.dean@uvahealth.org (M.D.); sherin.menachery@gmail.com (S.M.); aston.adam.greg@gmail.com (A.A.); 2 School of Medicine, Virginia Commonwealth University, Richmond, VA 23298, USA; banej2@vcu.edu (B.B.); alukooc@vcu.edu (O.A.); hangyw@vcu.edu (Y.H.); eomiller@wm.edu (E.M.); 3 Pauley Heart Center, Virginia Commonwealth University, Richmond, VA 23298, USA; jose.chuquin@vcuhealth.org (D.C.); jhjordan@vcu.edu (J.J.); 4 School of Public Health, Department of Biostatistics, Virginia Commonwealth University, Richmond, VA 23298, USA; xdeng@vcu.edu (X.D.); dbandyop@vcu.edu (D.B.); 5 Children’s National Department of Pediatric Cardiology, Washington, DC 20010, USA; utruong22@gmail.com; 6 Division of Pediatric Hematology Oncology, Virginia Commonwealth University, Richmond, VA 23298, USA; madhu.gowda@vcuhealth.org

**Keywords:** survivors of cancer diagnosed in childhood adolescence or young adulthood (C-AYAs), anthracyclines, dyslipidemia, hypertension, cardiovascular disease risk factors

## Abstract

**Background/Objectives**: Among survivors of cancer diagnosed in childhood, adolescence, or young adulthood (C-AYAs), cardiotoxic therapies combined with acquired cardiovascular risk factors (CVRFs) increase the risk for cardiovascular events. To our knowledge, no prior analysis has examined CVRFs among C-AYAs < 20 years old or compared CVRFs among younger and older C-AYAs. **Methods**: In this single-center study, individuals diagnosed with cancer at ≤39 years, treated with anthracycline-based chemotherapy (2010–2023), and with a post-treatment lipid panel and ≥2 post-treatment ambulatory blood pressure measurements were included. The CVRF prevalence was assessed among C-AYAs < 20 and ≥20 years old, using age-appropriate AAP and ACC/AHA guidelines. These prevalences were compared with the ICD-9/10 code prevalence. The prescription of medications with antihypertensive effects (MAHEs) and lipid-lowering therapy was assessed. **Results:** Among 276 C-AYAs, the median age was 28.1 years (IQR 18.1–38.3) at dyslipidemia screening and 29.3 (IQR 20.0–38.7) at hypertension screening. Dyslipidemia was present in 52.9% (146/276) and hypertension in 56.2% (155/276) of C-AYAs. C-AYAs < 20 years old had a high prevalence of dyslipidemia, 51.7% (45/87), and hypertension, 31.9% (29/91). CVRFs were frequently underdiagnosed, particularly dyslipidemia, among C-AYAs < 20 years old, with only 12.6% (11/87) having a diagnosis via the ICD code. C-AYAs < 20 years old with diagnoses of dyslipidemia and hypertension were significantly less likely to receive lipid-lowering therapy (2.2% vs. 14.9%) and trended toward less MAHEs (13.8% vs. 31.0%) compared to C-AYAs ≥ 20. **Conclusions**: Among C-AYAs treated with anthracyclines, dyslipidemia and hypertension were highly prevalent even at a young age (<20 years). Younger survivors with dyslipidemia and hypertension were less frequently prescribed lipid-lowering therapy or MAHEs.

## Simple Summary

Survivors of childhood, adolescent, and young adult-onset cancer (C-AYAs) represent a growing population at an increased risk for cardiovascular disease and mortality. Cardiovascular disease risk factors (CVRFs) increase this risk for cardiovascular disease in a synergistic fashion among survivors treated with anthracyclines. However, the data characterizing the prevalence of CVRFs—particularly among C-AYAs under 20 years of age—remains limited. In a cohort of C-AYAs treated with anthracyclines, we observed a high prevalence of dyslipidemia (51.7%) and hypertension (31.9%) among those less than 20 years old, indicating an early onset of CVRFs. Despite these findings, the prescription data demonstrated that younger C-AYAs were less likely than their older peers to be treated with lipid-lowering and antihypertensive therapy. These findings underscore the need for the proactive screening and management of CVRFs, beginning at an early age.

## 1. Introduction

More than 85% of individuals diagnosed with cancer before the age of 40 become long-term survivors [1]. Compared with non-cancer cohorts, survivors of childhood, adolescent, and young adult-onset cancer (C-AYAs) experience an increased risk for cardiovascular events and mortality persisting for decades after cancer diagnosis [2,3]. Modifiable cardiovascular risk factors (CVRFs) are known to act in an adverse synergistic fashion with cancer therapies to increase the risk for cardiovascular disease (CVD) among C-AYAs [2,4,5].

The presence of CVRFs in childhood and early adulthood increases the risk for CVD later in life [6]. Screening for these risk factors is recommended in survivorship guidelines [7,8,9]. However, CVRFs are often underdiagnosed among younger individuals [10,11,12,13]. To improve CVD outcomes among C-AYAs, it is crucial to understand the prevalence of modifiable CVRFs and rates of underdiagnosis earlier in survivorship. The data examining the dyslipidemia and hypertension prevalence among diverse populations of C-AYAs is limited. Among C-AYAs < 20 years old, no prior analysis has established the prevalence of CVRFs. Chow et al. utilized the Childhood Cancer Survivor Study (CCSS) to demonstrate a higher burden of dyslipidemia and hypertension among survivors of childhood cancer (CCS) as well as a greater risk for the undertreatment of these risk factors when compared with the general population [5]. However, this cohort did not include individuals < 20 years of age. A separate analysis of adolescent/young adult survivors (AYAs) in the Kaiser Permanente Southern California Network reported incidence rate ratios of 1.31 for dyslipidemia and 1.37 for hypertension compared to a matched non-cancer cohort. In this analysis, however, diagnosis relied upon the International Classification of Diseases (ICD) code rather than measured values, and the majority of individuals were in the third decade of life [14].

To our knowledge, this is the first analysis to use measured values to define the hypertension and dyslipidemia prevalence among C-AYAs < 20 years old, with a comparison to older C-AYAs. Further, the analysis examines the underdiagnosis and undertreatment of CVRFs among C-AYAs < 20 years old relative to older C-AYAs.

## 2. Materials and Methods

Individuals diagnosed with cancer at an age < 40 years, treated with systemic anthracycline or anthraquinone chemotherapy between 2010 and 2023 at Virginia Commonwealth University (VCU) Health Center, and who underwent guideline-recommended CVRF screening at our institution were retrospectively identified [7,8]. CVRF screening was defined as the presence of a post-treatment lipid panel and complete blood pressure data (as defined below). Exclusion criteria included death prior to remission. Demographic data, cancer history, most recent post-treatment smoking status, insurance status at diagnosis, body mass index (BMI) at diagnosis and within 3 months of most recent lipid panel, post-treatment prescription of lipid-lowering therapy (atorvastatin, pravastatin, rosuvastatin, simvastatin, fluvastatin, lovastatin, pitavastatin, ezetimibe, alirocumab, or evolocumab) and medications with antihypertensive effects (MAHE, beta-blocker, calcium channel blocker, alpha-2 agonist, alpha-1 blocker, angiotensin-converting enzyme inhibitor, angiotensin receptor blocker, angiotensin receptor neprilysin inhibitor, thiazide diuretic, loop diuretic, potassium sparing diuretic, or direct vasodilator) within 1 year of most recent post-treatment lipid panel or blood pressure (BP) reading, and vital status were abstracted from VCU medical records, cancer registry, billing claims, and Navigant death data via collaboration with VCU Massey Bioinformatics Core. Calculated doxorubicin isotoxic equivalents were based on the Children’s Oncology Group (COG) Long-Term Follow-Up Guidelines Version 6 [7]. Individuals were determined to have been diagnosed with dyslipidemia or hypertension if a corresponding ICD-9/10 code was identified in the medical record at any timepoint pre- or post-cancer diagnosis. This study was approved by the Institutional Review Board at VCU.

### 2.1. Dyslipidemia

Dyslipidemia via lipid panel was determined to be present if one or more measured lipids were abnormal based upon age-appropriate guidelines. Individuals included in the analysis had either (1) complete lipid panel data or (2) in the setting of incomplete lipid data at least one of the available values was abnormal. Most recent post-treatment fasting or non-fasting lipid panel was collected in accordance with current American Heart Association (AHA)/American College of Cardiology (ACC) guidelines [15]. Age at time of collection determined whether lipid panels were analyzed according to American Academy of Pediatrics (AAP) (<20 years) or AHA/ACC guidelines (≥20 years) [9,15]. AAP guidelines define abnormal values as low-density lipoprotein (LDL) ≥ 130, high-density lipoprotein (HDL) < 40, and triglyceride (TG) ≥ 100 (0–9 years old)/≥130 (10–19 years old), and total cholesterol (TC) ≥ 200 [9]. AHA/ACC guidelines define abnormal values as LDL ≥ 160, HDL < 40, and TG > 150 [15].

### 2.2. Hypertension

Based on congruence between pediatric and adult BP definitions, up to ten most recent ambulatory BP measurements recorded after completion of all cancer therapy and at ≥13 years of age were included. Individuals with complete BP data had at least two post-treatment BP readings within twelve months. Hypertension was defined as two or more readings within twelve months with systolic BP ≥ 130 or diastolic BP ≥ 80 in accordance with AAP/AHA/ACC hypertension guidelines [16,17].

### 2.3. Statistical Analysis

The prevalence of dyslipidemia and hypertension was determined using the most recently recorded blood pressures and lipid measurements and ICD-9/10 codes. The prevalences were compared among C-AYAs < 20 years old and C-AYAs ≥ 20 years old. The dyslipidemia and hypertension prevalence among C-AYAs < 20 years old and ≥20 years old was then examined based on sex (male, female), race (White, Black, Other), doxorubicin isotoxic equivalents dose (≤250 mg/m^2^, >250 mg/m^2^), insurance status (private, Medicaid/Medicare/not insured, VA/Tricare, unknown), post-treatment BMI (healthy weight, overweight, and obese—defined by percentile for those <20 years old [18,19]), and most recent tobacco use (former smoker, current smoker, never smoker). The dyslipidemia analysis included BP and vice versa. To validate ICD code prevalence, medical records for 50 patients without diagnoses of dyslipidemia and hypertension via ICD code were manually reviewed to screen for documentation of the presence of these CVRFs. Chi-square analysis, Fisher’s exact test, Wilcoxon rank-sum test, and Cochran–Mantel–Haenszel test (CMH) were utilized as appropriate. McNemar’s test was utilized to assess underdiagnosis by comparing the prevalence of CVRFs via measured values and ICD code. Regression models assessed odds ratios for dyslipidemia and hypertension via measured values and ICD codes, while adjusting for age, race, doxorubicin isotoxic equivalent dose, insurance status, BMI category, tobacco use, and hypertension or dyslipidemia status. The regression analysis used complete case analysis; only three participants were excluded due to missing smoking status. Among those diagnosed with dyslipidemia, the proportion that received a prescription for lipid-lowering therapy from our health system was determined. A similar analysis was performed to assess prescription of MAHE among those diagnosed with hypertension. All statistical analysis was performed using SAS 9.4 (Statistical Analysis System, version 9.4).

## 3. Results

Between 2010 and 2023, 276 C-AYAs were treated with systemic anthracycline chemotherapy at our institution and underwent a cardiovascular risk assessment, including lipid panels and blood pressure measurements. Most individuals identified as White (n = 145, 52.5%) or Black (n = 108, 39.1%) races (Table 1). The most common cancer types were leukemia, 40.6% (n = 112), Hodgkin lymphoma, 18.5% (n = 51), and non-Hodgkin lymphoma, 17.4% (n = 48). In total, 32.6% (n = 90) received an anthracycline dose of >250 mg/m^2^ doxorubicin isotoxic equivalents, and 26.1% (n = 72) had a history of chest radiation.

Among older and younger C-AYAs, the race, sex, and insurance status did not significantly differ. Younger C-AYAs were more likely to have a diagnosis of leukemia (48.3%, n = 42), while older C-AYAs had a higher prevalence of Hodgkin, 19.0% (n = 36), and Non-Hodgkin lymphoma, 18.5% (n = 35), as well as breast cancer, 12.7% (n = 24). Older C-AYAs had significantly higher total doxorubicin isotoxic equivalents, 240.0 mg/m^2^ (IQR 150.0–300.0) versus 150 mg/m^2^ (IQR 75.0–200.0), and were more likely to have received chest radiation, 30.2% (n = 57). Older C-AYAs had a significantly higher BMI than the younger cohort, 27.4 kg/m^2^ (IQR 23.2–33.2) versus 21.7 kg/m^2^ (IQR 19.1–28.7), and were more likely to be current (7.9%, n = 15; 2.3%, n = 2) and former smokers (28.6%, n = 54; 2.3%, n = 2).

### 3.1. Dyslipidemia

**Figure 1:**
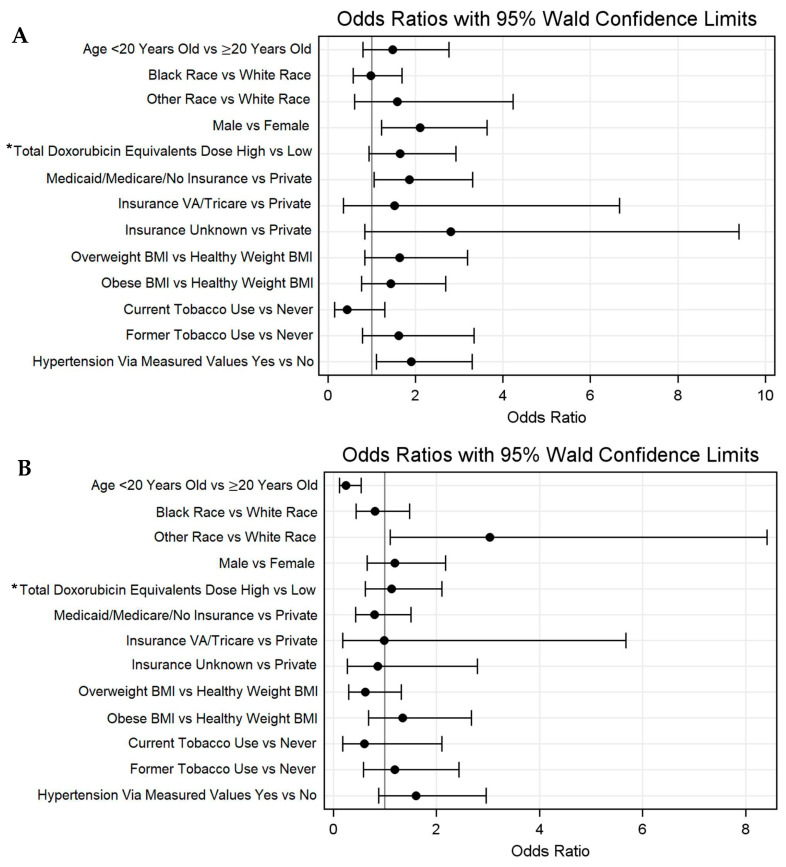
Odds ratios with 95% Walds confidence limits for dyslipidemia via (**A**) lipid panel and (**B**) ICD-9/10 codes. * High > 250 mg/m^2^; low ≤ 250 mg/m^2^.

The median age at the most recent lipid panel was 28.1 years (IQR 18.1–38.3), with 31.5% of individuals (n = 87) being < 20 years old. The median time from the cancer diagnosis to the lipid panel was 3.9 (1.9–7.0) years. Among the total population, 52.9% (n = 146) met the criteria for dyslipidemia via lipid panels. HDL (35.9%) and TGs (34.7%) were the most commonly abnormal lipids (Supplementary Table S1). The regression analysis identified several variables associated with increased odds of dyslipidemia via lipid panels, including male sex (OR = 2.1, 95% CI 1.2–3.7), Medicaid/Medicare/Not Insured (OR = 1.9, 95% CI 1.1–3.3), and patients with hypertension via measured values (OR = 1.9, 95% CI 1.1–3.3). The odds of dyslipidemia via measured values were not associated with age or a non-White race. The high total doxorubicin equivalent dose (OR = 1.7, 95% CI 0.9–2.9) and past tobacco use (OR = 1.6, 0.8–3.4) demonstrated non-significant trends toward a higher odds of dyslipidemia (Figure 1A, Supplementary Table S2). Regarding dyslipidemia via the ICD code, a younger age was associated with decreased odds of dyslipidemia (OR = 0.2, 95% CI 0.1–0.5) (Figure 1B).

**Figure 2:**
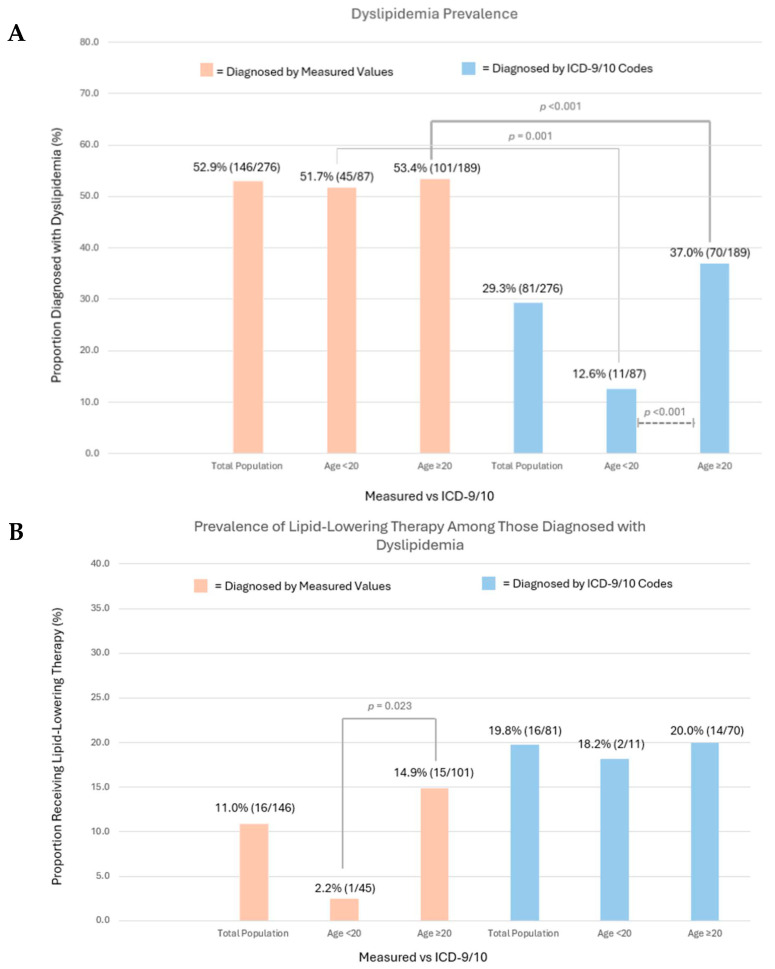
(**A**) Dyslipidemia prevalence via measured values and ICD-9/10 codes in the total population and by age group (<20 years, ≥20 years). Chi-square analysis compared prevalence rates among age groups, with significant results depicted (dashed lines). McNemar’s test compared measured value vs. ICD code prevalence, with significant results depicted (solid lines). (**B**) Prescription of lipid-lowering therapy among those diagnosed with dyslipidemia, reported among the total population and by age group (<20 years, ≥20 years). Chi-square analysis compared prevalence of therapy among age groups, with significant results depicted (solid lines).

A substantial burden of dyslipidemia was noted among both younger and older C-AYAs, at 51.7% (n = 45) and 53.4% (n = 101), respectively (Figure 2A, Supplementary Table S3). No significant difference in the dyslipidemia prevalence between the two age groups was observed after stratifying by race, sex, total doxorubicin equivalent dose, insurance status, BMI, tobacco use history, and hypertension history. Among both younger and older C-AYAs, high dyslipidemia prevalence rates were noted among C-AYAs who were male, had exposure to total doxorubicin isotoxic equivalent doses > 250 mg/m^2^, and had public/no insurance. The prevalence of abnormal TG and HDL values did not vary significantly by age.

The underdiagnosis of dyslipidemia was common as the ICD-9/10 code prevalence among the total population was 29.3% (n = 81). Among the entire cohort, significant discrepancies between measured and ICD code prevalence rates for dyslipidemia were observed among individuals with public/no insurance (<20 years old measured values 55.6% [n = 15], ICD-9/10 11.1% [n = 3]; ≥20 years old measured values 65.3% [n = 47], ICD-9/10 36.1% [n = 26], *p* < 0.001). Age influenced underdiagnosis, and among those <20 years old, only 12.6% (n = 11) of individuals had an ICD-9/10 code for dyslipidemia, compared to 51.7% (n = 45) via measured values (*p* < 0.001). Among those ≥20 years old, 37.0% (n = 70) had an ICD-9/10 code for dyslipidemia compared to 53.4% (n = 101) via measured values (*p* = 0.001). Our manual chart review found that only 4.0% of patients without an ICD code for dyslipidemia had a provider note documenting the diagnosis, suggesting that underdiagnosis, rather than variations in clinical documentation practices, was the primary driver of our finding.

Among the total population, only 11.0% of C-AYAs diagnosed with dyslipidemia via lipid panels were prescribed lipid-lowering therapy by a provider at our institution. When stratified by age, those ≥20 years old were significantly more likely to receive lipid-lowering therapy compared with those <20 years old (14.9% [n = 15], 2.2% [n = 1], *p* = 0.023). Among C-AYAs with the ICD-9/10 code for dyslipidemia, a significant difference in the prescription of lipid-lowering therapy among the age groups was not appreciated (<20 years old 18.2% [n = 2], ≥20 years old 20.0% [n = 14], *p* = 0.999) (Figure 2B).

### 3.2. Hypertension

**Figure 3:**
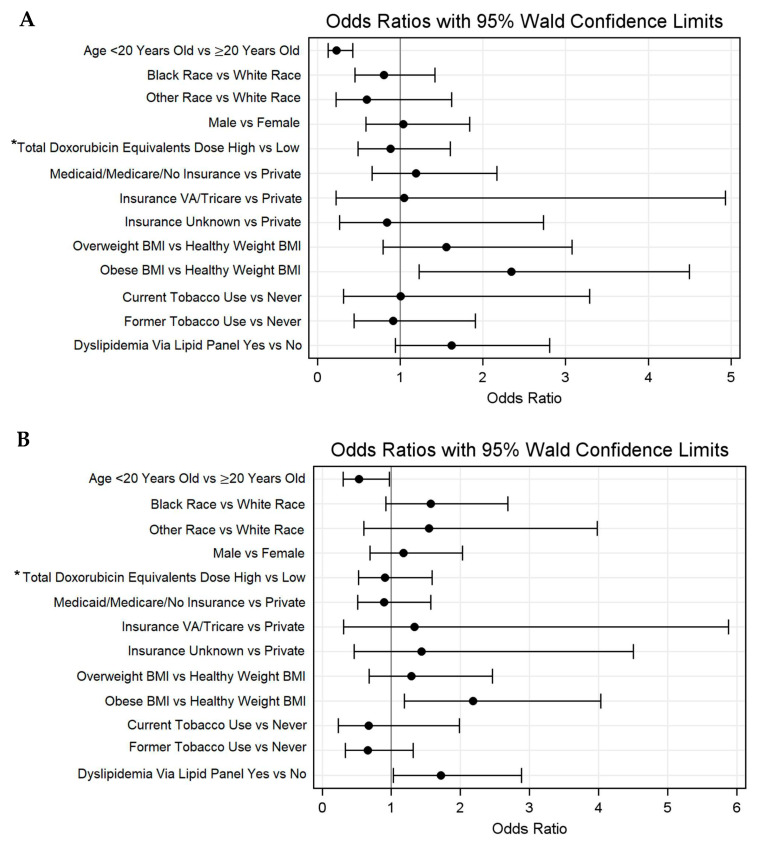
Odds ratios with 95% Walds confidence limits for hypertension via (**A**) measured values and (**B**) ICD-9/10 codes. * High > 250 mg/m^2^ and low ≤ 250 mg/m.

The median age at the most recent blood pressure assessment was 29.0 years (IQR 20.0–38.7), with 33.0% of individuals being (n = 91) <20 years old. The median time from the cancer diagnosis to the most recent blood pressure was 5.7 (IQR 3.0–6.8) years. Among the total population, 56.2% (n = 155) of individuals met the criteria for hypertension via recorded BP values. The regression analysis demonstrated that a younger age was associated with decreased odds of hypertension via measured values (HR = 0.2, 0.1–0.4). An obese BMI was associated with increased odds of hypertension via measured values (HR = 2.3, 1.2–4.5). Race, sex, and total doxorubicin equivalent dose, and tobacco use were not associated with increased odds of hypertension via measured values. An overweight BMI (HR = 1.6, 0.8–3.1) and dyslipidemia via lipid panels (HR = 1.6, 0.9–2.8) each demonstrated non-significant trends toward higher odds of hypertension via measured values (Figure 3A, Supplementary Table S4). Regarding dyslipidemia via the ICD code, a younger age was associated with decreased odds of dyslipidemia (HR = 0.5, 0.3–0.98). An obese BMI (HR = 2.2, 1.2–4.0) and a history of dyslipidemia via lipid panels (HR = 1.7, 1.0–2.9) were associated with increased odds of hypertension via the ICD code (Figure 3B).

**Figure 4:**
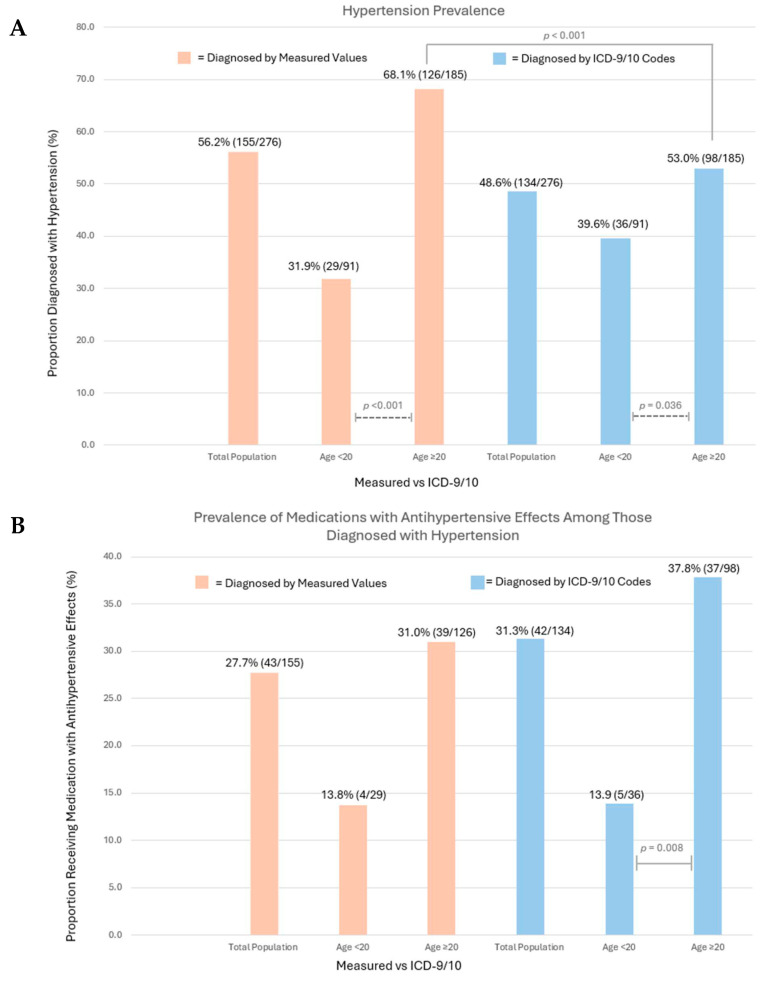
(**A**) Hypertension prevalence via measured values and ICD-9/10 codes in the total population and by age group (<20 years, ≥20 years). Chi-square analysis compared prevalence rates among age groups, with significant results depicted (dashed lines). McNemar’s test compared measured value vs. ICD code prevalence, with significant results depicted (solid lines). (**B**) Prescription of medications with antihypertensive effects (MAHEs) among individuals diagnosed with hypertension, reported among the total population and by age group (<20 years, ≥20 years). Chi-square analysis compared prevalence of therapy among age groups, with significant results depicted (solid lines).

A substantial burden of hypertension was noted among both younger and older C-AYAs, 31.9% (n = 29) and 68.1% (n = 126) (*p* < 0.001), respectively (Figure 4A, Supplementary Table S5). In total, 33.3% (n = 92/276) of the population carried a diagnosis of both hypertension and dyslipidemia via measured values, with 17.3% (16/92) of these individuals being <20 years old.

Among those <20 years old, subgroups with the highest hypertension prevalence rates included public/no insurance, 40.7% (n = 11), overweight and obese BMI (38.5% [n = 5], 38.5% [n = 11]), and those with dyslipidemia, 37.8% (n = 17). Among those ≥20 years old, hypertension prevalence rates exceeded 70% in those with exposure to total doxorubicin isotoxic equivalent doses > 250 mg/m^2^, 70.4% [n = 50], public/no insurance, 70.8% (n = 51), obese BMI, 79.7% (n = 51), a history of tobacco use, 70.4% (n = 50), and dyslipidemia, 74.3% (n = 75).

Hypertension was underdiagnosed with a prevalence, via the ICD-9/10 code, of 48.6% (n = 134). Among C-AYAs ≥ 20 years of age, only 53.0% (n = 98) of individuals had an ICD-9/10 code for hypertension, compared to 68.1% (n = 126) via measured values (*p* < 0.001). Our manual chart review found that only 4.0% of patients without an ICD code for hypertension had a provider note documenting the diagnosis.

Among the total population, only 27.7% of C-AYAs diagnosed with hypertension via measured values were prescribed MAHEs by a provider at our institution. When stratified by age, the prescription of the MAHE was less prevalent among the younger cohort, with a strong non-significant trend observed (13.8% [n = 4] in those <20 years vs. 31.0% [n = 39] in those ≥20 years, *p* = 0.062), likely limited by the small sample size of the younger hypertensive group (n = 29). Among C-AYAs with a diagnosis via the ICD-9/10 code, the younger subgroup was significantly less likely to receive MAHEs (<20 years old 13.9% [n = 5], ≥20 years old 37.8% [n = 37], *p* = 0.008) (Figure 4B).

## 4. Discussion

The present study leveraged a cohort of young C-AYAs treated with anthracyclines (31.5% <20 years of age) to examine the prevalence, diagnosis, and treatment of CVRFs. Among this population, (1) dyslipidemia and hypertension were highly prevalent and commonly underdiagnosed, (2) individuals < 20 years old exhibited a high burden of dyslipidemia (51.7%) and hypertension (31.9%), and (3) survivors with dyslipidemia or hypertension who were <20 years of age were less commonly prescribed lipid-lowering therapy or MAHEs relative to older survivors.

Chao et al. and Chow et al. have demonstrated a high burden of CVRFs among C-AYAs who are >20 years of age [5,14]. Among older C-AYA cohorts, a greater attained age is associated with the premature development of CVRFs [20,21]. To our knowledge, our work is the first to incorporate an age-specific analysis, focused on C-AYAs < 20 years old, suggesting an early onset of CVRFs among C-AYAs. This finding is of significant clinical importance, as the synergistic effects of cancer therapy toxicity and CVRFs amplify long-term CVD risks, emphasizing the need for early diagnosis and aggressive CVRF modification [2]. Strong links between childhood-onset CVRFs and adult-onset CVD, along with evidence that the CVD risk increases with the duration of dyslipidemia and hypertension, further underscore the importance of early CVRF modifications among a population at an increased risk for CVD [6,22,23]. Despite these links, the data suggests that younger patients are less likely to receive a diagnosis of hypertension than their older counterparts, despite regular follow-ups, suggesting that lower clinical suspicion may contribute to underdiagnosis [12].

Despite the high prevalence of CVRFs identified through objective measures, the comparison with the ICD code data revealed that underdiagnosis was common, supporting Chow et al.’s finding of significant CVRF underdiagnosis among CCSs [5]. Notably, our study shows that among individuals < 20 years old, there was a 39% discrepancy between the lipid panel and ICD code prevalence, suggesting that the early onset of dyslipidemia among C-AYAs is often underrecognized. The regression analysis highlighted that C-AYAs with public/no insurance had nearly twice the odds of dyslipidemia compared with those who were privately insured. The underdiagnosis of dyslipidemia was significant amongst this group, particularly amongst C-AYAs < 20 years old, at 45%. Insurance status is a marker of socioeconomic status (SES) that reflects underlying structural drivers of inequity and health disparities. A recent scientific statement published by the AHA highlighted disparities in cardio-oncology and cited limited insurance and access to follow-up as a driver of disparities [24]. Amongst C-AYAs, a lower SES has been linked to CVRF development and CVD mortality [25,26]. The biological and social mechanisms underlying these associations are likely multifactorial. One mechanism of particular relevance in C-AYAs—who often experience enduring psychological stress related to cancer diagnosis and treatment—is the interplay between a lower SES and psychological distress. A lower SES has been associated with heightened amygdalar activity, a marker of chronic stress, with downstream effects including upregulated arterial inflammation, vasoconstriction, and endothelial dysfunction [27,28]. This in combination with a greater burden of CVRFs highlights an important opportunity for increased CVRF screening among those with lower SESs, beginning at a young age.

The COG Long-Term Follow-Up Guidelines, together with current National Comprehensive Cancer Network (NCCN) and International Guideline Harmonization Group (IGHG) recommendations, support more frequent screening for dyslipidemia and hypertension among C-AYAs [7,8,29]. The COG guidelines specifically recommend annual blood pressure assessments for individuals exposed to anthracyclines and a lipid evaluation every two years for those with a history of abdominal or total body irradiation [7]. Similarly, the NCCN guidelines endorse cardiovascular risk factor screening in survivors who received combined anthracycline and radiation therapy or higher-dose radiation to the chest or abdomen [8]. The IGHG guidelines further recommend tailoring screening based on the intensity of the cardiotoxic exposure, family history, and the presence of comorbid conditions [29]. These guidelines differ from current United States Preventative Task Force guidelines, which recommend hypertension screening every 3–5 years among those <40 years old and periodic lipid screening beginning at age 40 [30,31]. With varying access to survivorship clinics throughout the United States, our results support interventions to increase the awareness of current guidelines and increased frequency of CVRF screening among C-AYAs in the primary care setting beginning at an early age. While the COG and NCCN guidelines focus on screening for dyslipidemia among survivors who have received radiation, in our analysis, the prevalence of dyslipidemia among those with >250 mg/m^2^ total doxorubicin equivalent doses approached 60% and was independent of the age group. While prospective analyses are needed to establish a relationship between the cumulative anthracycline dose and dyslipidemia risk, these results suggest that limiting dyslipidemia screening among older and younger C-AYAs to those with a radiation history may lead to underdiagnosis.

The high prevalence of dyslipidemia and hypertension among our cohort as well as the early age of onset calls into question the utility of current ACC/AHA guidelines for dyslipidemia and hypertension management in the C-AYA population [16,32]. These guidelines utilize risk scoring to influence treatment decisions regarding dyslipidemia and hypertension. Recent hypertension and dyslipidemia guidelines utilize the Predicting Risk of Cardiovascular Disease Events (PREVENT) score—which incorporates more variables than the prior Atherosclerotic Cardiovascular Disease (ASCVD) risk score. Analyses to date have demonstrated several mechanisms that might explain the high prevalence of CVRFs among C-AYAs, including epigenetic age acceleration, chronic inflammation, and therapy-induced changes in lipid metabolism. Our findings highlight the importance of further defining these mechanisms to help address the CVRF prevalence among this population and highlight that risk scores derived from the general population may not be applicable to C-AYAs [32,33,34,35,36,37].

Chow et al. have demonstrated that a large proportion of CCSs meeting the clinical criteria for CVRFs were likely to be undertreated [5]. The prescription data from our cohort demonstrates that only 11% and 28% of individuals meeting the criteria for dyslipidemia and hypertension were prescribed lipid therapy or MAHE, with a disproportionate undertreatment of dyslipidemia (2.2% vs. 14.9%) and hypertension (13.8% vs. 31.0%) among C-AYAs < 20 years of age relative to those ≥20. These results likely highlight downstream effects of limited CVRF screening combined with a lack of treatment guidelines among C-AYAs. However, given the primary lipid abnormalities among both age groups included HDL and TG, these findings may additionally reflect the current ACC/AHA recommendations amongst the general population, emphasizing lifestyle modifications for hypertriglyceridemia management [15]. Similarly, NCCN and COG guidelines emphasize lifestyle interventions, such as referrals to dieticians, when CVRFs are identified [7,8]. Our results highlight the need for future work that examines not only the prescription of therapy—whether that is lifestyle measures or medical therapy—but also longitudinal data looking at the control of risk factors among this population.

The dyslipidemia and hypertension prevalence among our population of C-AYAs exceeds national averages among the general population, with only 25.2% and 3.7–6.6% of adolescents reported to have dyslipidemia and hypertension, respectively, as well as previously reported estimates among cancer survivors [5,38,39,40,41]. Several factors may explain this high burden of CVRFs. To begin, the analysis utilized objective measures for both the dyslipidemia and hypertension prevalence rather than ICD codes [14]. To our knowledge, this study is the first to assess the hypertension prevalence among C-AYAs based on serial BP measurements, providing additional diagnostic opportunities. Among both age groups, dyslipidemia and hypertension prevalence rates were higher among C-AYAs with public/no insurance compared to those who were privately insured. As highlighted above, with the SES being tied to CVRF development and CVD mortality, we hypothesize that the high prevalence of these risk factors among our cohort is reflective of our catchment area, in which poverty rates exceed national averages [42].

Analyses of measured lipids determined that the dyslipidemia among C-AYAs was primarily driven by abnormalities in HDL and TG. A higher TG/HDL ratio has been demonstrated in several studies to predict cardiovascular outcomes. Further, it has been linked with highly atherogenic lipid phenotypes, including higher remnant lipoprotein cholesterol and LDL density [43,44]. These findings suggest the importance of addressing this lipid phenotype, particularly in a population at a higher risk for CVD later in life. The results also suggest an overlap between hypertension and dyslipidemia diagnoses. Notably, abnormalities in BP, TG, and HDL are three of the five diagnostic criteria for metabolic syndrome, and prior analyses have shown an increased prevalence of metabolic syndrome among survivors compared to cancer-free peers [45,46]. Of note, among our younger cohort, despite a high prevalence of hypertension and dyslipidemia, the median BMI was normal, and an abnormal BMI was not associated with increased odds of dyslipidemia. Similarly, in the Chow et al. study CCSs were less likely to be obese compared to the general population [5]. It is possible that this lack of an abnormal BMI contributes to the underdiagnosis of CVRFs among C-AYAs and that the metabolic abnormalities seen in C-AYAs may be independent of central adiposity. Analyses examining the use of the BMI in CCSs have estimated that the BMI underestimates obesity by 52%, suggesting that a normal BMI does not always reflect a healthy body composition or metabolic profile amongst this population [47]. As highlighted above, current IGHG recommendations include a CVRF assessment in C-AYAs with additional comorbidities. Importantly, our results suggest that it may be reasonable to screen C-AYAs independent of BMI.

### Limitations

Limitations of this study include its retrospective, single-center design and relatively small cohort size. Our cohort consisted of 73% AYAs. This population accounts for only 5% of all cancer cases in the United Sates and remains under-represented in research, underscoring the importance of single-center analyses [1,48]. VCU Health is a tertiary care center, which may have led to selection bias, as patients with multiple medical comorbidities are more likely to be referred. Although the overall medical complexity may be higher in a tertiary care population, the relative medical complexity is likely similar across subgroups and any such bias would likely have affected both age groups similarly, minimizing the impact on comparisons between age groups. Thus, the findings still suggest that younger patients remain at meaningful risk. To allow for serial blood pressure measurements in accordance with ACC/AHA/AAP guidelines, the median time from the cancer diagnosis to the complete blood pressure data collection was 1.8 years longer than the time from the cancer diagnosis to the lipid data collection, which may influence the comparability of the prevalence of these two CVRFs [16,17]. BP readings obtained in the clinic setting may have been impacted by the white coat effect; however, untreated white coat hypertension is associated with an increase in adverse cardiovascular events, and current ACC/AHA guidelines support the use of office readings when data is available from several visits [49]. Masked hypertension may have also been undetected. The prevalence based on the ICD code may have been underestimated, as diagnoses might not have always had corresponding ICD codes in the electronic health records. However, the manual chart review demonstrated that this was uncommon. The optimal control of CVRFs among patients may have resulted in patients not meeting objective dyslipidemia and hypertension criteria, potentially underestimating rather than overestimating the CVRF prevalence. The treatment status was based on prescriptions for lipid-lowering therapy or MAHE at VCU Health, which may have missed prescriptions from out-of-network providers. However, given patients were followed longitudinally at VCU Health, where the CVRF screening was performed, we hypothesize that the majority of the CVRF management occurred at VCU Health. In addition, prior analyses did not report medication data or utilized self-reports for prescriptions, which may be subject to response bias [5,14]. The use of a binary cut-off of 20 years may oversimply the complex relationship between aging and CVRF risks. However, this classification was selected intentionally, given individuals < 20 years old have been under-represented in the literature, and this age represents the transition point between AAP and ACC/AHA guidelines [9,15,16,17]. In addition, an age of 20 represents a commonly used transition point between adolescence and young adulthood in oncology and survivorship research, including in the American Cancer Society reporting of cancer demographics [50].

## 5. Conclusions

Among a younger cohort of C-AYAs treated with anthracyclines, dyslipidemia and hypertension were common with prevalences of 51.7% and 31.9%, respectively, among those <20 years old. A comparison of measured values with the ICD-9/10 code prevalence revealed that underdiagnosis was common, particularly the underdiagnosis of dyslipidemia among those <20 years old. Individuals < 20 years old with diagnoses of dyslipidemia and hypertension were less likely to receive lipid-lowering therapy or MAHE compared to those ≥20 years old. These results highlight the limitations of current screening practices and the potential opportunity for the timely initiation of lifestyle modifications and medical therapy for CVRF modification among younger C-AYAs. We hope that this work provides a foundation for future large-scale, multi-center analyses that will provide definitive guidance to mitigate the substantial CVD burden in this population.

## Figures and Tables

**Table 1:** Baseline demographic and patient variables of interest, stratified by age at timing of lipid panel. IQR = interquartile range.

Variable	Total Cohort n (%)	<20 Years Old, n (%)	≥20 Years Old, n (%)	*p*-Value
	N = 276	N = 87	N = 189	
**Race**				0.3836
Black	108 (39.1)	31 (35.6)	77 (40.7)	
White	145 (52.5)	46 (52.9)	99 (52.4)	
Other	23 (8.3)	10 (11.5)	13 (6.9)	
**Sex**				0.0718
Female	152 (55.1)	41 (47.1)	111 (58.7)	
Male	124 (44.9)	46 (52.9)	78 (41.3)	
**Cancer Type**				0.0071
Leukemia	112 (40.6)	42 (48.3)	70 (37.0)	
Hodgkin Lymphoma NOS	51 (18.5)	15 (17.2)	36 (19.0)	
Non-Hodgkin’s Lymphoma	48 (17.4)	13 (14.9)	35 (18.5)	
Other Hematopoietic	3 (1.1)	1 (1.1)	2 (1.1)	
Sarcoma	29 (10.5)	10 (11.5)	19 (10.1)	
Renal	1 (0.4)	1 (1.1)	0 (0.0)	
Breast	24 (8.7)	0 (0.0)	24 (12.7)	
Ovarian	1 (0.4)	0 (0.0)	1 (0.5)	
Rectal	1 (0.4)	0 (0.0)	1 (0.5)	
Thyroid	1 (0.4)	1 (1.1)	0 (0.0)	
Other Cancer	5 (1.8)	4 (4.6)	1 (0.5)	
**Total Doxorubicin Isotoxic Equivalents Dose, mg/m^2^, Median (IQR)**	200.0 (125.0–300.0)	150.0 (75.0–200.0)	240.0 (150.0–300.0)	<0.0001
**Total Doxorubicin Isotoxic Equivalents Dose**				0.0017
High (>250)	90 (32.6)	17 (19.5)	73 (38.6)	
Low (≤250)	186 (67.4)	70 (80.5)	116 (61.4)	
**History of Chest Radiation**				0.0132
Yes	72 (26.1)	15 (17.2)	57 (30.2)	
No	188 (68.1)	63 (72.4)	125 (66.1)	
Missing	16 (5.8)	9 (10.3)	7 (3.7)	
**Prescribed Lipid-Lowering Therapy**				0.0182
Yes	22 (8.0)	2 (2.3)	20 (10.6)	
No	254 (92.0)	85 (97.7)	169 (89.4)	
**Prescribed Medication with Antihypertensive Effects**				0.0006
Yes	57 (20.7)	8 (8.8)	49 (26.5)	
No	219 (79.3)	83 (91.2)	136 (73.5)	
**Smoking Status**				<0.0001
Current	17 (6.2)	2 (2.3)	15 (7.9)	
Former	56 (20.3)	2 (2.3)	54 (28.6)	
Never	200 (72.5)	83 (95.4)	117 (61.9)	
Unknown	3 (1.1)	0 (0.0)	3 (1.6)	
**Insurance Status at Diagnosis**				0.1709
Medicaid/Medicare/Not Insured	99 (35.9)	27 (31.0)	72 (38.1)	
Private	153 (55.4)	55 (63.2)	98 (51.9)	
VA/Tricare	8 (2.9)	3 (3.4)	5 (2.6)	
Unknown	16 (5.8)	2 (2.3)	14 (7.4)	
**Variable**	**Median, (IQR)**	**<20 Years Old,** **Median, (IQR)**	**≥20 Years Old** **Median, (IQR)**	***p*** **-value**
BMI at Time of Lipid Panel, kg/m^2^	25.6 (21.5–31.5)	21.7 (19.1–28.7)	27.4 (23.2–33.2)	<0.0001
Age at Diagnosis, Years	23.7 (14.6–33.4)	10.7 (5.9–14.4)	29.8 (23.4–35.8)	<0.0001
Age at Most Recent Lipid Panel, Years	28.1 (18.1–38.3)	15.4 (13.3–17.8)	34.2 (27.7–40.5)	<0.0001
Time from Diagnosis to Lipid Panel, Years	3.9 (1.9–7.0)	4.1 (1.9–7.1)	3.9 (1.8–6.9)	0.6670
Age at Most Recent Blood Pressure, Years	29.0 (20.0–38.7)	-	-	-
Time from Diagnosis to Most Recent Blood Pressure, Years	5.7 (3.0–6.8)	-	-	-

## Data Availability

Data are available on request due to restrictions; this will need to be reviewed by the VCU Health IRB for approval.

## Supplementary Materials

The following supporting information can be downloaded at https://www.mdpi.com/article/10.3390/cancers18010012/s1, Table S1: Prevalence of abnormal values for measured fats including low-density lipoprotein (LDL), high-density lipoprotein (HDL), total cholesterol (TC), and triglycerides (TG). Presented as total population and stratified by age group; Table S2: Odds ratios with 95% Walds confidence limits for dyslipidemia via lipid panel and ICD-9/10 codes; Table S3: Prevalence of dyslipidemia among those aged <20 and ≥20 years old, further stratified by race, sex, age, total doxorubicin equivalents dose, insurance status, tobacco use, BMI, and diagnosis of hypertension. Chi-square analysis compared prevalence among sub-groups, McNemar’s compared lipid panel vs ICD code prevalence; Table S4: Odds ratios with 95% Walds confidence limits for hypertension via measured values and ICD-9/10 codes; Table S5: Prevalence of hypertension among those aged <20 and ≥20 years old, further stratified by race, sex, age, total doxorubicin equivalents dose, insurance status, tobacco use, BMI, and diagnosis of hypertension. Chi-square analysis compared prevalence among sub-groups, McNemar’s compared lipid panel vs ICD code prevalence.

## Abbreviations

The following abbreviations are used in this manuscript:

C-AYAs	Survivors of Childhood, Adolescent, and Young Adult-Onset Cancer
CVRFs	Cardiovascular Risk Factors
CVD	Cardiovascular Disease
CCSS	Childhood Cancer Survivor Study
CCSs	Childhood Cancer Survivors
AYAs	Survivors of Adolescent and Young Adult-Onset Cancer
ICD	International Classification of Diseases
VCU	Virginia Commonwealth University
BMI	Body Mass Index
MAHE	Medication with Antihypertensive Effects
BP	Blood Pressure
COG	Children’s Oncology Group
AHA	American Heart Association
ACC	American College of Cardiology
AAP	American Academy of Pediatrics
LDL	Low-Density Lipoprotein
HDL	High-Density Lipoprotein
TG	Triglyceride
TC	Total Cholesterol
CMH	Cochran–Mantel–Haenszel Test
SES	Socioeconomic Status
NCCN	National Comprehensive Cancer Network
IGHG	International Guideline Harmonization Group
