# Supplementary material for: Cardiovascular Risk Factors Among Younger and Older C-AYA Cancer Survivors Treated with Anthracyclines: A Single-Center Analysis
Source: Cancers (Basel). 2025 Dec 19;18(1):12. doi: 10.3390/cancers18010012 (PMC12785066; doi:10.3390/cancers18010012)
Supplement: Supplementary file 1 [file cancers-18-00012-s001.zip › cancers-3944678-supplementary.pdf]

Measured Fat	Abnormal, n (%)	Chi-Square, p-value
LDL	21 (8.1)	
<20	5 (6.2)	0.4485
≥20	16 (8.9)	
HDL	94 (35.9)	
<20	28 (34.6)	0.7674
≥20	66 (36.5)	
TC		
<20	3 (3.7)	-
TG	96 (34.7)	
<20	27 (31)	0.3751
≥20	69 (36.5)	

Supplemental Table S1. Prevalence of abnormal values for measured fats including low-density lipoprotein (LDL), high-density lipoprotein (HDL), total cholesterol (TC), and triglycerides (TG). Presented as total population and stratified by age group.

Odds Ratio Estimates and Wald Confidence Intervals						
	Dyslipidemia Via Measured Values			Dyslipidemia via ICD-9/10 Code		
Effect	Estimate	95% Confidence Interval	p-value	Estimate	95% Confidence Interval	p-value
Age <20 Years Old vs ≥20 Years Old	1.48	0.788-2.781	0.2229	0.246	0.11-0.549	0.0006
Black Race vs White Race	0.982	0.564-1.709	0.9474	0.805	0.436-1.486	0.4879
Other Race vs White Race	1.588	0.595-4.239	0.3559	3.036	1.095-8.420	0.0328
Male vs Female	2.107	1.215-3.653	0.0079	1.19	0.647-2.190	0.5751
Total Doxorubicin Equivalents Dose, High vs Low	1.653	0.93-2.938	0.0869	1.135	0.608-2.117	0.6911
Medicaid/Medicare/No Insurance vs Private	1.864	1.048-3.315	0.0339	0.802	0.424-1.519	0.4988
Insurance VA/Tricare vs Private	1.522	0.347-6.674	0.5774	0.991	0.173-5.684	0.9917
Insurance Unknown vs Private	2.808	0.838-9.406	0.0942	0.862	0.265-2.804	0.8046
Overweight BMI vs Healthy Weight BMI	1.638	0.838-3.205	0.1492	0.621	0.29-1.332	0.221
Obese BMI vs Healthy Weight BMI	1.434	0.761-2.703	0.2653	1.346	0.675-2.684	0.3989
Current Tobacco Use vs Never	0.438	0.147-1.306	0.1387	0.605	0.173-2.111	0.4303
Former Tobacco Use vs Never	1.621	0.783-3.355	0.193	1.19	0.58-2.445	0.635
Hypertension via Measured Values Yes vs No	1.904	1.094-3.313	0.0227	1.605	0.867-2.971	0.1319

Supplemental Table S2. Odds ratios with 95% Walds confidence limits for dyslipidemia via lipid panel and ICD-9/10 codes.

	Dyslipidemia Prevalence Via Lipid Panel n (%), Chi-square P-value		Dyslipidemia Prevalence Via ICD-9/10 Code n (%), Chi-Square P-Value		McNemar's Test P-Value: Recorded Value vs ICD Code Prevalence
**Age**					
<20 years	45 (51.7)	0.7909	11 (12.6)	<0.0001	<0.0001
≥20 years	101 (53.4)		70 (37.0)		0.0010
**Race**					
Black, age <20 years	14 (45.2)	0.2586	4 (12.9)	0.0501	0.0039
Black, age ≥20 years	44 (57.1)		24 (31.2)		0.0012
White, age <20 years	27 (58.7)	0.2084	5 (10.9)	0.0007	<0.0001
White, age ≥20 years	47 (47.5)		38 (38.4)		0.1699
Other, age <20 years	4 (40.0)	0.0721	2 (20.0)	0.0464	0.3173
Other, age ≥20 years	10 (76.9)		8 (61.5)		0.4795
**Sex**					
Female, age <20 years	18 (43.9)	0.8999	5 (12.2)	0.0043	0.0016
Female, age ≥20 years	50 (45.0)		40 (36.0)		0.1489
Male, age <20 years	27 (58.7)	0.4564	6 (13.0)	0.0026	<0.0001
Male, age ≥20 years	51 (65.4)		30 (38.5)		0.0010
**Total Doxorubicin Equivalents Dose**					
High (>250 mg/m^2), age <20 years	10 (58.8)	0.7483	2 (11.8)	0.0557	0.0114
High (>250 mg/m^2), age ≥20 years	46 (63.0)		26 (35.6)		0.0012
Low (≤250 mg/m2), age <20 years	35 (50.0)	0.7324	9 (12.9)	0.0002	<0.0001
Low (≤250 mg/m2), age ≥20 years	55 (47.4)		44 (37.9)		0.1235
**Insurance Status**					
Medicaid/Medicare/No Insurance, age <20 years	15 (55.6)	0.3732	3 (11.1)	0.0149	0.0005
Medicaid/Medicare/No Insurance, age ≥20 years	47 (65.3)		26 (36.1)		0.0004
Private, age <20 years	26 (47.3)	0.6855	8 (14.5)	0.0025	0.0004
Private, age ≥20 years	43 (43.9)		37 (37.8)		0.3865
VA/Tricare, age <20 years	2 (66.7)	0.4652	0 (0)	0.2059	-
VA/Tricare, age ≥20 years	2 (40.0)		2 (40.0)		1.0000
Unknown, age <20 years	2 (100.0)	0.3081	0 (0)	0.3081	-
Unknown, age ≥20 years	9 (64.3)		5 (35.7)		0.0455
**Post-Treatment Body Mass Index**					
Healthy weight, age <20 years (<85th percentile)	22 (44.0)	0.6260	5 (10.0)	0.0002	0.0004
Healthy weight, age ≥20 years (<25.0 kg/m^2)	33 (48.5)		28 (41.2)		0.3532
Overweight, age <20 years (85th-95th percentile)	7 (53.8)	0.6316	1 (7.7)	0.1273	0.0143
Overweight, age ≥20 years (≥25.0 - <30 kg/m^2)	33 (61.1)		15 (27.8)		0.0020
Obese, age <20 years (>95th percentile)	16 (61.5)	0.4666	4 (15.4)	0.0108	0.0005
Obese, age ≥20 years (≥30 kg/m^2)	34 (53.1)		28 (43.8)		0.2393
**Tobacco Use**					

Current Smoker, age <20 years	1 (50.0)	0.7872	0 (0)	0.4036	-
Current Smoker, age ≥20 years	6 (40.0)		4 (26.7)		0.5271
Former Smoker, <20 years	1 (50.0)	0.5819	0 (0)	0.2467	-
Former Smoker, age ≥20 years	37 (68.5)		22 (40.7)		0.0071
Never smoker, age <20 years	43 (51.8)	0.5825	11 (13.3)	0.0003	<0.0001
Never smoker, age ≥20 years	56 (47.9)		42 (35.9)		0.0390
Unknown, age <20 years	0 (0)	-	0 (0)	-	-
Unknown, age ≥20 years	2 (66.7)		2 (66.7)		1.0000
**Hypertension (Via Recorded Values)**					
Yes, age <20 years	16 (55.2)	0.6110	5 (17.2)	0.0189	0.0076
Yes, age ≥20 years	76 (60.3)		51 (40.5)		0.0019
No, age <20 years	26 (41.9)	0.5413	6 (9.7)	0.0022	<0.0001
No, age ≥20 years	28 (47.5)		19 (32.2)		0.0833

Supplemental Table S3. Prevalence of dyslipidemia among those aged <20 and ≥20 years old, further stratified by race, sex, age, total doxorubicin equivalents dose, insurance status, tobacco use, BMI, and diagnosis of hypertension. Chi-square analysis compared prevalence among sub-groups, McNemar's compared lipid panel vs ICD code prevalence.

**Odds Ratio Estimates and Wald Confidence Intervals**						
	**Hypertension Via Measured Values**			**Hypertension via ICD-9/10 Code**		
Effect	Estimate	95% Confidence Interval	p-value	Estimate	95% Confidence Interval	p-value
Age <20 Years Old vs ≥20 Years Old	0.231	0.123-0.434	<0.0001	0.535	0.293-0.977	0.0417
Black Race vs White Race	0.803	0.451-1.428	0.4546	1.569	0.914-2.691	0.1021
Other Race vs White Race	0.599	0.22-1.628	0.3151	1.543	0.597-3.988	0.3709
Male vs Female	1.037	0.583-1.846	0.9013	1.178	0.682-2.035	0.5572
Total Doxorubicin Equivalents Dose, High vs Low	0.886	0.487-1.611	0.6919	0.910	0.520-1.595	0.7426
Medicaid/Medicare/No Insurance vs Private	1.193	0.654-2.176	0.5658	0.895	0.508-1.578	0.702
Insurance VA/Tricare vs Private	1.048	0.222-4.935	0.953	1.333	0.302-5.883	0.7041
Insurance Unknown vs Private	0.844	0.26-2.735	0.7771	1.437	0.458-4.511	0.5347
Overweight BMI vs Healthy Weight BMI	1.56	0.79-3.082	0.2005	1.288	0.671-2.470	0.4471
Obese BMI vs Healthy Weight BMI	2.347	1.224-4.500	0.0102	2.185	1.181-4.040	0.0127
Current Tobacco Use vs Never	1.007	0.308-3.294	0.9906	0.671	0.226-1.990	0.4718
Former Tobacco Use vs Never	0.915	0.437-1.916	0.8145	0.659	0.328-1.323	0.2406
Dyslipdemia via Lipid Panel Yes vs No	1.622	0.937-2.810	0.0843	1.717	1.02-2.892	0.0421

Supplemental Table S4. Odds ratios with 95% Walds confidence limits for hypertension via measured values and ICD-9/10 codes.

	Hypertension Prevalence Via Measured Values n (%), Chi-square P-value		Hypertension Prevalence Via ICD-9/10 Code n (%), Chi-Square P-Value		McNemar's Test P-Value: Recorded Value vs ICD Code Prevalence
**Age**					
<20 years	29 (31.9)	<0.0001	36 (39.6)	0.0361	0.2087
≥20 years	126 (68.1)		98 (53.0)		0.0005
**Race**					
Black, age <20 years	11 (33.3)	0.0008	15 (45.5)	0.1611	0.2482
Black, age ≥20 years	51 (68.0)		45 (60.0)		0.2008
White, age <20 years	15 (31.3)	<0.0001	16 (33.3)	0.1066	0.7815
White, age ≥20 years	68 (70.1)		46 (47.4)		0.0002
Other, age <20 years	3 (30.0)	0.2528	5 (50.0)	0.8548	0.4142
Other, age ≥20 years	7 (53.8)		7 (53.8)		1.0000
**Sex**					
Female, age <20 years	15 (35.7)	0.0006	17 (40.5)	0.1767	0.5930
Female, age ≥20 years	73 (66.4)		58 (52.7)		0.0163
Male, age <20 years	14 (28.6)	<0.0001	19 (38.8)	0.1125	0.2253
Male, age ≥20 years	53 (70.7)		40 (53.3)		0.0093
**Total Doxorubicin Equivalents Dose**					
High (>250 mg/m^2), age <20 years	5 (26.3)	0.0005	5 (26.3)	0.0201	1.0000
High (>250 mg/m^2), age ≥20 years	50 (70.4)		40 (56.3)		0.0330
Low (≤250 mg/m2), age <20 years	24 (33.3)	<0.0001	31 (43.1)	0.2983	0.1936
Low (≤250 mg/m2), age ≥20 years	76 (66.7)		58 (50.9)		0.0055
**Insurance Status**					
Medicaid/Medicare/No Insurance, age <20 years	11 (40.7)	0.0058	16 (59.3)	0.3451	0.1317
Medicaid/Medicare/No Insurance, age ≥20 years	51 (70.8)		35 (48.6)		0.0025
Private, age <20 years	15 (25.9)	<0.0001	17 (29.3)	0.0022	0.6171
Private, age ≥20 years	64 (67.4)		52 (54.7)		0.0339
VA/Tricare, age <20 years	1 (25.0)	0.1573	2 (50.0)	1.0000	0.5637
VA/Tricare, age ≥20 years	3 (75.0)		2 (50.0)		0.3173
Unknown, age <20 years	2 (100.0)	0.2416	1 (50.0)	0.6963	-
Unknown, age ≥20 years	8 (57.1)		9 (64.3)		0.5637
**Post-Treatment Body Mass Index**					
Healthy weight, age <20 years ($<85^{th}$ percentile)	11 (22.0)	<0.0001	18 (36.0)	0.4661	0.0522
Healthy weight, age ≥20 years (<25.0 kg/m^2)	41 (60.3)		29 (42.6)		0.0105
Overweight, age <20 years (85th-95th percentile)	5 (38.5)	0.0610	4 (30.8)	0.1376	0.7055

Overweight, age ≥20 years (≥25.0 - <30 kg/m^2)	36 (66.7)		29 (53.7)		0.1266
Obese, age <20 years (>95th percentile)	10 (38.5)	0.0001	15 (57.7)	0.8831	0.0956
Obese, age ≥20 years (≥30 kg/m^2)	51 (79.7)		38 (59.4)		0.0067
**Tobacco Use**					
Current Smoker, age <20 years	0 (0)	-	0 (0)	-	-
Current Smoker, age ≥20 years	12 (70.6)		8 (47.1)		0.0039
Former Smoker, <20 years	0 (0)	0.0364	1 (50.0)	1.0000	0.2733
Former Smoker, age ≥20 years	38 (70.4)		27 (50.0)		0.0455
Never smoker, age <20 years	29 (32.6)	<0.0001	35 (39.3)	0.0279	0.2733
Never smoker, age ≥20 years	73 (65.8)		61 (55.0)		0.0455
Unknown, age <20 years	0 (0)	-	0 (0)	-	-
Unknown, age ≥20 years	3 (100.0)		2 (66.7)		-
**Dyslipidemia (Via Lipid Panel)**					
Yes, age <20 years	17 (37.8)	<0.0001	24 (53.3)	0.7276	0.0896
Yes, age ≥20 years	75 (74.3)		57 (56.4)		0.0035
No, age <20 years	10 (23.8)	0.0001	13 (31.0)	0.1156	0.4054
No, age ≥20 years	53 (60.2)		40 (45.5)		0.0124

Supplemental Table S5. Prevalence of hypertension among those aged <20 and ≥20 years old, further stratified by race, sex, age, total doxorubicin equivalents dose, insurance status, tobacco use, BMI, and diagnosis of hypertension. Chi-square analysis compared prevalence among sub-groups, McNemar's compared lipid panel vs ICD code prevalence.
